# Prevalence of Newly Diagnosed End-Stage Renal Disease Patients in a Tertiary Hospital of Central Nepal, Chitwan: A Descriptive Cross-sectional Study

**DOI:** 10.31729/jnma.4971

**Published:** 2021-01-31

**Authors:** Madhav Ghimire, Shreeju Vaidya, Hari Prasad Upadhyay

**Affiliations:** 1 Department of Nephrology, College of Medical Sciences Teaching Hospital, Bharatpur, Nepal; 2 Department of Community Medicine, College of Medical Sciences Teaching Hospital, Bharatpur, Nepal

**Keywords:** *diabetes mellitus*, *end stage kidney disease*, *prevalence*

## Abstract

**Introduction:**

End-stage renal disease patients are in rising trend globally, and they have been found to occur predominantly in developing countries. Many studies have been published before, within and across the countries, to know the clinicodemographic profile of end-stage renal disease patients. However, no such studies were done in Chitwan, Nepal. This study's main objective was to find the prevalence of newly diagnosed end-stage renal disease patients.

**Methods:**

A hospital-based descriptive cross-sectional study was carried out in the Department of Nephrology from May 2016 to April 2019. Convenient sampling was done, and all the consecutive new end-stage renal disease patients were included in the study. The ethical approval was taken from the Institutional Review Committee (reference number. 2016/COMSTH/IRC/042). The prevalence and demographic profile of new end-stage renal disease patients were studied. The data were analyzed with appropriate statistical tools.

**Results:**

A total of 250 new end-stage renal disease patients were found among 2200 admitted patients. The prevalence of new end-stage renal disease was found to be 250 (11.36%). Out of 250 patients, males were 156 (62.4%), and females were 94 (37.6%). The mean age was 49.6±15.5 years. The commonest cause of the incident end-stage renal disease was Type 2 Diabetes mellitus 89 (35.6%).

**Conclusions:**

The prevalence of new end-stage renal disease was found to be quite high. The commonest cause of the incident end-stage renal disease was Type 2 Diabetes Mellitus.

## INTRODUCTION

End-stage renal disease (ESRD) is a major public health problem worldwide and is associated with significant morbidity and mortality.^1,2^ New ESRD patients are rising globally, and the increase has been found to occur predominantly in developing countries.^3,4^

We had realized that the new ESRD patients were increasing in our center. The precise data regarding the true prevalence of ESRD patients lack in Nepal, a retrospective multicentric study done by Hada et al.^5^ revealed that the incidence of ESRD had increased gradually from 3.4 per million populations (pmp) in 1990 to 11.89 pmp in 1999 with an average annual incidence of 6 pmp. However, there were no previous studies regarding the prevalence and clinicodemographic profile of new ESRD patients from Chitwan, Nepal.

Therefore, we thought of doing a study to know the prevalence of new ESRD patients among 2200 admitted renal patients in our center.

## METHODS

A hospital-based descriptive cross-sectional study was carried out in the Department of Nephrology of the college of medical science from May 2016 to April 2019. The ethical approval was taken from the college's Institutional Review Committee (reference number. 2016/COMSTH/IRC/042), and written informed consents were taken from the patients. Convenient sampling was done, and all the consecutive new ESRD patients, irrespective of their age, sex, and renal diagnosis, were included in the study. A nephrologist made the clinical diagnosis of renal diseases with an experience of > 5 years in clinical Nephrology, and all the diagnoses were supported by relevant biochemistry, radiology, and pathology reports. The standard definitions were used to define the renal diagnosis as per the updated kidney disease improving global outcome (KDIGO) equivalent criteria, wherever applicable.

Sample size for this research was calculated by taking p and q as 50% with 95% CI and 7% margin of error. The sample size was calculated using the formula below,

$$\begin{array}{ll} \text{n} & ={\text{Z}}^{\text{2}}\text{pq}/{\text{e}}^{\text{2}}\\ & ={\left(1.96\right)}^{2}\times (0.5)\times (0.5)/{\left(0.07\right)}^{\text{2}}\\ & =196 \end{array}$$

By adding a 10% non-response rate, this research's actual sample size was 216, but this research was conducted among 250 patients.

The patient's demographic profile and cause of new ESRD were noted in the proforma. The data were then entered in the MS XP sheet and then were transferred to the SPSS version 20 (Chicago, IL, USA) program and were analyzed using appropriate statistical tools. The continuous variables were expressed as mean ± standard deviation (SD) and ratio. The categorical variables were expressed as frequency and percentage.

## RESULTS

Over 3 years, from May 2016 to April 2019, a total of 2200 patients were admitted to the Department of Nephrology. Of them, a total of 250 patients were new ESRD patients. So the prevalence of new ESRD was found to be 11.36%.

Out of 250 new ESRD patients, 156 (62.4%) were males, and 94 (37.6%) were females. The mean age of the patient was 49.6 years with a standard deviation of ±15.5 years. The minimum age was 17 years and the maximum 85 years. In terms of age distribution, 75 (30.0%) patients were of age < 40 years, and 175 (70%) were of age ≥40 years.

**Table 1:** Age and gender distribution of new ESRD patients (n = 250).

Age (in years)	Male n (%)	Female n (%)	Total n (%)
1-20	4 (57.1)	3 (42.9)	7 (2.8)
21-40	46 (67.7)	22 (32.3)	68 (27.2)
41-60	56 (53.9)	48 (46.1)	104 (41.6)
61-80	48 (69.6)	21 (30.4)	69 (27.6)
>80	2 (100)	-	2 (0.8)

The causes of new ESRD were as shown in table 2. Of the 250 new ESRD patients, kidney biopsies proved diagnosis were in six (2.4%) patients. Out of them, 4 (1.6%) were IgA nephropathy, one (0.4%) lupus nephritis (LN), and one (0.4%) membranous glomerulopathy (MGN).

**Table 2:** Causes of new ESRD patients (n = 250).

Etiology of new ESRD	n (%)
Type 2 Diabetes mellitus (T2DM)	89 (35.6%)
Chronic Glomerulonephritis (CGN)	86 (34.4%)
Hypertension (HTN)	60 (24.0%)
Obstructive Uropathy	6 (2.4%)
Autosomal dominant polycystic kidney disease	3 (1.2%)
Lupus Nephritis	2 (0.8%)
Neurogenic bladder	1 (0.4%)
Congenital anomalies of kidney urinary tract	1 (0.4%)
Acute Tubular Necrosis (ATN)	1 (0.4%)
Renal Calculus Disease (RCD)	1 (0.4%)
Total	250 (100%)

Out of 250 new ESRD patients, body mass index (BMI) < 18.5 Kg/m^2^ were seen in 22 (8.8%) patients, between 18.5-24.9 Kg/m^2^ were in 143 (57.2%), and ≥25 Kg/m^2^ were in 85 (34%) patients.

## DISCUSSION

The true burden of ESRD faced by Nepal remains unknown due to the limited access of chronic kidney disease (CKD) patients to healthcare facilities, lack of organized CKD management programs, and lack of national registries. Unlike most developed countries, Nepal has yet to develop a national registry of ESRD patients. Available data come from few tertiary referral centers and may therefore not represent the country as a whole. ESRD incidence is rising worldwide and even so in the developing world.^1–4^ To the best of our knowledge, this study was the first of its kind from Chitwan to know the prevalence and clinicodemographic profile of new ESRD patients. A total of 250 new ESRD were identified among 2200 admitted patients for 3 years, this makes the prevalence of new ESRD to be 11.36%, which is quite high for a center like ours, reflecting a high burden of ESRD in this region. Out of 250 new ESRD patients, 62.4% were males, and 37.6% were females making a male preponderance with an M: F ratio of 1:6, which was concordant with the studies done in India.^6,7^ Similar observations of male dominance with an M: F ratio of ~2:1 were seen in dialysis outcome and practice pattern study (DOPPS)^8^ and Indian study.^9,10^ This dominance of males over the female may reflect the socio-dynamic influence of our society, where a treatment privilege goes to males. It might be because the males are inherently predisposed to develop kidney diseases. This area of research needs multicentric genetic studies with a uniform protocol.

The patient's mean age was 49.6 years with a standard deviation of ±15.5 years, which was comparable to a study done by Al-Wakeel et al^11^ where the mean age of presentation was 53.7 years. However, the mean age was lesser as compared to Okinawa Dialysis Study (OKIDS) Registry (55.9 years),^12^ and united states renal data system (USRDS) 2008 data (64.4 years).^13^ Majority of the patients, 70.0%, were of age ≥ 40 years, highlighting that ESRD is a middle-age disease (average age of presentation was around 50 years).

The commonest cause of new ESRD in our study was T2DM, 35.6%, followed by CGN, 34.4%, and HTN 24.0%. There is considerable variation in the causes of ESRD within and across the countries. The etiologic patterns seen in our study were consistent with previous studies.^9,14^ and the CKD registry of India.^10^ However, Sathyan et al.^15^ found CGN 51.0% and diabetic nephropathy 22.0% as the most common aetiologies of CKD. Chaudhari et al.,^16^ Jha et al.^17^ and Parsi et al.^18^ found diabetic nephropathy, hypertensive nephropathy and chronic glomerulonephritis as the most common etiology of CKD. In a recent population-based survey, diabetes was the cause of CKD in 41% of cases.^19^ Similarly in another study^20^ involving several hospitals in India found that 29% of CKD subjects had diabetic nephropathy. Many of these studies, including our highlight the dominance of diabetic nephropathy as the major cause of ESRD worldwide.

This dominance of diabetes as the major cause may be due to the worldwide rising diabetes mellitus.^21,22^

The majority of the patients, 91.2% in the new ESRD group (n=250), had normal to overweight BMI, making undernutrition a less prevalent problem 8.8%. This might be because most of the patients were above their dry weight, to begin with, due to volume overload, which might have falsely overestimated the BMI and underrepresented the true malnutrition prevalence.

There were few limitations in our study. First, this was an observational study without a control group hence;, all the inherent limitations of an observational study were there in the study. This was also a single-center study, so the results might not be generalizable to the whole region and the country, highlighting the need for multicentric studies with a uniform protocol.

## CONCLUSIONS

The prevalence of new ESRD was 11.36%, which is quite high for a center like ours, reflecting a high burden of ESRD in this region, and the commonest cause of incident ESRD was T2DM. These data suggest that ESRD is a growing problem in our region. There is an urgent need to expand the dialysis service centers and kidney transplant programs to accommodate the new and increasing ESRD patients.
